# The Influence of Contextual Aspects in Talent Development: Interaction Between Relative Age and Birthplace Effects in NBA-Drafted Players

**DOI:** 10.3389/fspor.2021.642707

**Published:** 2021-03-22

**Authors:** Nuno Leite, Jorge Arede, Ximing Shang, Julio Calleja-González, Alberto Lorenzo

**Affiliations:** ^1^Research Centre in Sports Sciences, Health Sciences and Human Development (CIDESD), Vila Real, Portugal; ^2^Department of Sports, Exercise and Health Sciences, University of Trás-os-Montes and Alto Douro, Vila Real, Portugal; ^3^Department of Physical Education and Sport, Faculty of Education and Sport, University of Basque Country, Vitoria-Gasteiz, Spain; ^4^Facultad de Ciencias de la Actividad Física y el Deporte, Universidad Politécnica de Madrid, Madrid, Spain

**Keywords:** environmental factors, talent development, date of birth, place of birth, interaction, selection, basketball

## Abstract

The aims of this study were two-fold: (1) to inspect separately for the relative age and birthplace effects for players selected in the National Basketball Association (NBA) draft; (2) to explore the interaction among these factors and analyse this interaction in players' career performance. The database was obtained from the official records of the players (*n* = 1,738), who were selected during the annual editions of the NBA Draft from 1990 to 2019. The participants' date of birth was analyzed according to the month of birth and divided into four quartiles. The place of birth was compared to the distribution of the general population' places of birth based on different communities' sizes. Chi-square analysis were used to determine if the relative age and birthplace of the players drafted differed in any systematic way from official census population distributions. Cluster analysis and standardized residuals were calculated to analyse the interaction among the contextual factors and the players' career performance. The data revealed that early-born players (Q1 and Q2) were over-represented. Moreover, players born in smaller cities (<100,000) were over-represented. The interaction analysis revealed that the players born in the bigger communities relate mainly with relatively younger players, and clusters that correspond to players born in smaller communities integrated the relatively older players. No differences were found in the players' career performance. Researchers, coaches and practitioners should be aware of the interaction between contextual factors to help nurture the development of sport talent regardless of age-related issues or communities' size.

## Introduction

A growing core of scientific research has shown that contextual factors play a key role in talent development (Baker et al., 2009; Rees et al., 2016; Williams et al., 2020). In particular, the role of contextual variables such as the date of birth (Castillo et al., 2019; De la Rubia et al., 2020a), and the place of birth (Rossing et al., 2015; Pennell et al., 2017; Wattie et al., 2018) have been consistently associated with improved chances of attaining elite sport.

The biased distribution of the dates of birth, known as the relative age effect (RAE), has been confirmed in several studies in the field of sports sciences and other domains, such as in academic performance (for references see Nakata et al., 2017). In that way, evidence confirm this phenomenon, in which individuals born earlier in the selection year relative to a predetermined cut-off date (e.g., January 1–December 31) are often overrepresented compared to those born later in the same selection year (Fumarco et al., 2017; please see De la Rubia et al., 2020a). In this sense, relatively older athletes are systematically associated with higher stature and better aerobic capacity, endurance and speed, what provides them with a competitive advantage in physical performance (Rada et al., 2018). Related to physical advantages, critical in childhood and adolescence, the relatively older athletes are generally considered to have greater potential and consequently with an increased likelihood of being selected to play and practice in a more challenging environment, and to be supervised by more experienced coaches, what may eventually lead to improved feedback mechanism and competitiveness (Musch and Grondin, 2001; Bezuglov et al., 2019). In contrast, relatively late-born athletes are more unlikely to obtain similar early opportunities, which will inevitably make their road to success more difficult (Arede et al., 2019a).

Scientific evidence in the literature has shown that contextual factors such as the place of birth (known as birthplace effects where the subject was born and grew up during the developmental years, Lidor et al., 2014) also can influence an athlete's likelihood of elite sporting attainment (Côté et al., 2006; MacDonald et al., 2009b; Imtiaz et al., 2014; Turnnidge et al., 2014; Ishigami, 2016; Wattie et al., 2018; Kaida and Kitchen, 2020). In that way, mixed results have been reported for the birthplace effects. For example, early studies (Côté et al., 2006) revealed that athletes born in cities of less than 500,000 inhabitants were more likely to play for professional leagues than athletes born in larger cities. Researchers reported that higher developmental opportunities may be associated with smaller cities, including improved mobility and safety conditions for play and practice, cultural and closer personal relationship between athletes and coaches (Davids and Baker, 2007). Nevertheless, recent studies showed different results as larger cities were just as effective in producing elite athletes in smaller cities (e.g., Schorer et al., 2010). The authors highlighted the fact that bigger cities can offer better-designed and equipped sporting facilities (e.g., arenas, fields, and swimming pools) and more experienced coaches (see Baker et al., 2009; Schorer et al., 2010). Despite such assumptions, the extent to which these trends apply to other sports and sport development systems in other countries remains to be determined. Though some authors have already identified some inconsistencies in the birthplace definition (Rossing et al., 2015), fundamentally due to the differences found among the place of birth, the athlete's place of development, the first club, and the inaccurate interpretation that may arise from this misconception, the fact is that birthplace studies are still scarce and further contributions can be potentially decisive to improve the process of talent identification and development.

Over the past few years, some studies have examined the RAE and the birthplace effects with the same group of athletes (Côté et al., 2006; Baker et al., 2009; MacDonald et al., 2009a; Lidor et al., 2014; Turnnidge et al., 2014; Ishigami, 2016). However, most of these studies analyzed these contextual factors on limited geographical areas. In fact, there is an important gap to cover in the scientific literature that could be bridged in comparing youth sport policies and developmental systems. For instance, the development of high-level athletes in European countries is mainly based on club teams, while in the USA, they emerge from the school system. Moreover, while prior studies have confirmed the influence of these factors in a number of different sports in various countries, they have not demonstrated how these aspects interact (Bruner et al., 2011) and affect the chances of playing in a professional league like the NBA (*National Basketball Association*). The odds of a pre-high school player being chosen to play at a professional level in the NBA are intrinsically low, and only a small part of these players is selected through the NBA Draft (Koz et al., 2012). This is competitive event, where the league franchises obtain the professional rights of players who do not yet have a contract with any team in the competition. A total of 60 players are selected each year and the order of selection of these players is stipulated by the lottery draft that takes in account the teams' final balance of victories and defeats from the last NBA season.

To the best of our knowledge, this is the first study dedicated in examining the long-term effect of both contextual factors, in the NBA career performance. Thus, the aims of this study were two-fold: (1) to inspect separately for the RAE and birthplace effects for USA players and for players born in the European countries; (2) to explore the interaction among these contextual factors and the long-term effect in the players' career performance. Drawing upon existing literature in sport sciences, it was hypothesized that the RAE and the birthplace effects influence the likelihood of being selected to play in the NBA; and, that the interaction among these factors exist and that it can also be observed in the players' career performance.

## Methods

### Participants

**Figure 1:**
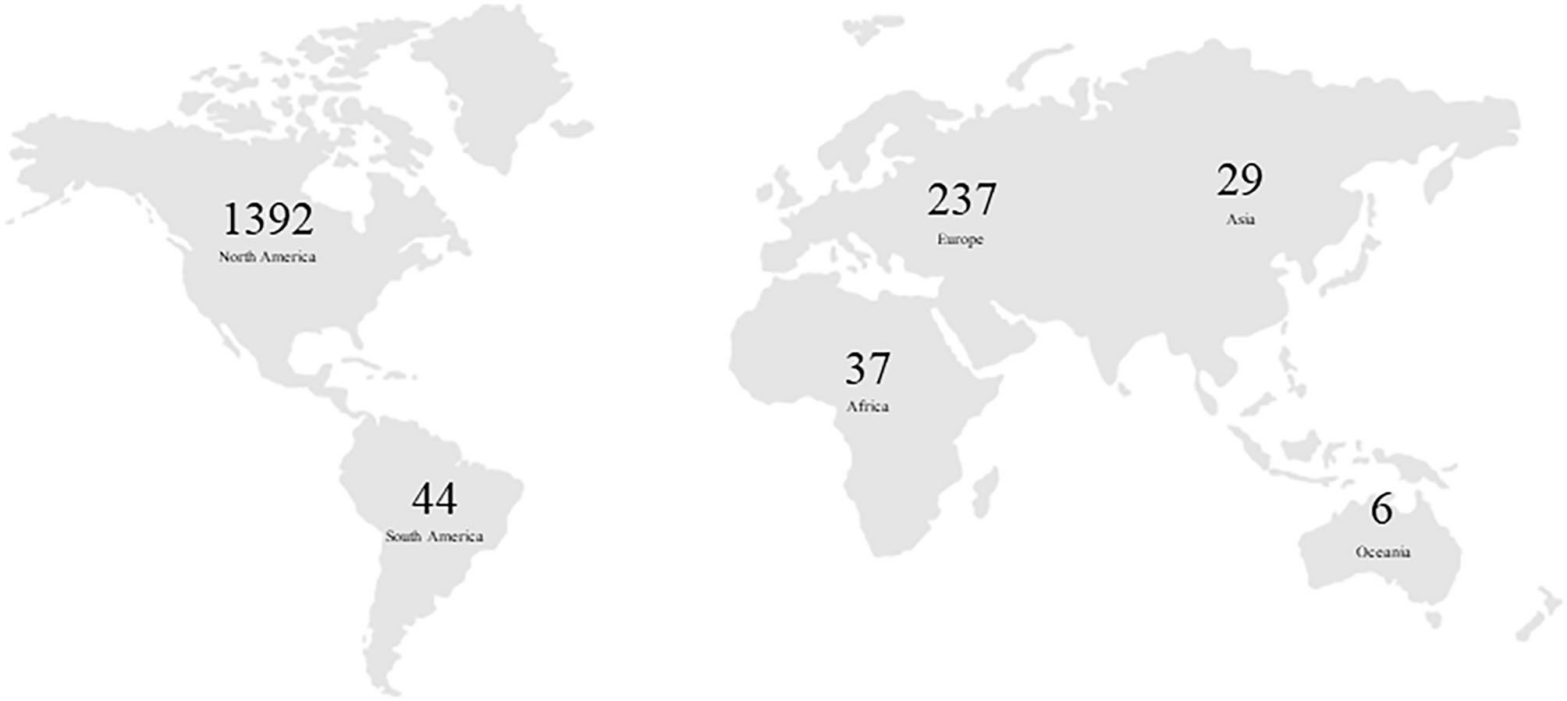
Number of players drafted per continent (between 1990 and 2019).

The database was obtained from the records of all players (*n* = 1,738), selected during the annual editions of the NBA Draft, from 1990 to 2019. The database included players born in 67 different countries, 1,389 players born in the USA, 237 born in the European countries, 37 in African countries, 44 in South American countries, and finally 29 in Asian countries (Figure 1 and Table 1).

**Table 1:** Number of players drafted per country (between 1990 and 2019).

**Country**	**# Players**
USA	1,389
France	30
Serbia	26
Bosnia-Herzegovina	17
Spain	16
Lithuania	14
Croatia, Germany, Slovenia	12
Greece, Turkey	11
Brazil, Nigeria, Ukraine	10
Montenegro	9
England, Senegal	8
China, Russia	7
Italy, Latvia	6
Czechoslovakia, Israel	5
Argentina, Australia, Belgium, Congo, Georgia, Switzerland, Sudan, Sweden, Dominican Republic	4
Canada, Finland, Ghana, Haiti, Guadeloupe, Netherlands, Mali, Poland, Puerto Rico	3
Egypt, Jamaica, New Zealand, Romania, Trinidad and Tobago, Zaire	2
Angola, Austria, Belarus, Cape Verde, Central African Republic, Denmark, Estonia, Guinea, Hungary, India, Iran, Japan, Korea, Uzbekistan, Venezuela, Taiwan, South Africa, Saint Vincent and the Grenadines, Mexico, Luxembourg	1

The players who were selected in the NBA Draft but failed in accumulating a significant experience in the league, i.e., never played in any NBA team or did not completed 1 full and consecutive season (minimum 82 games) were not considered for analysing the *second aim of this study*, i.e., the interaction among the contextual factors and the players' career performance. The present study was approved by the institutional research ethics committee (UID04045/2020) and conformed to the recommendations of the Declaration of Helsinki, with the Fortaleza actualization (Hellmann et al., 2014).

### Procedure

**Figure 2:**
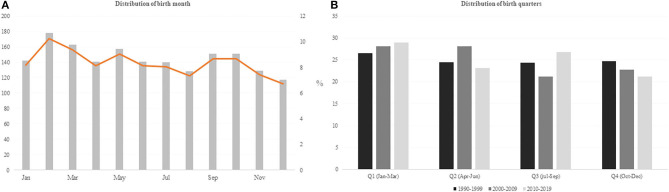
Distribution of **(A)** birth months (left) and **(B)** birth quarters according to the decades of the NBA draft (right).

Official players' data collection was completed in two consecutive steps. First, the date and place of birth of the players selected to the NBA Draft, were collected from the Basketball reference website (https://www.basketball-reference.com/). Second, individual career stats accumulated in the NBA were collected form the official league website (https://www.nba.com/). The participants' date of birth was first analyzed according to the month of birth. The cut-off date was January 1^st^. Thus, the year was divided into four quartiles: Q1, January 1^st^–March 31^st^; Q2, April 1^st^–June 30^th^; Q3, July 1^st^–September 30^th^; and Q4, October 1^st^–December 31^st^ (Figure 2).

The place of birth was compared to the distribution of the general population' places of birth according to different city or communities' sizes categorization (Côté et al., 2006), into 1 of 8 census population categories: (1) more than 5,000,000 million of inhabitants; (2) between 2,500,000 and 4,999,999; (3) 1,000,000–2,499,000; (4) 500,000–999,999; (5) 250,000–499,999; (6) 100,000–249,999; (7) 50,000–99,999; (8) less than 50,000 inhabitants.

To investigate the *first hypothesis* and considering the considerable number of players born in European countries drafted by NBA teams and simultaneously the interest of comparing the RAE and birthplace across countries and continents, we conducted a separate analysis for players born in the USA and born in the European countries. However, the singularity of each European country registration system, the methods of defining demographic maps as well as, the particularities of the territorial organization limited a more comprehensive analysis of the interaction among the RAE and the birthplace also for the players born in European countries (i.e., the *second hypothesis*). This procedure was also supported by the lack of consistency of the evidence found in several studies examining the birthplace effects in European born players in comparison with, for example USA (Baker et al., 2009; Lidor et al., 2010, 2014; Schorer et al., 2010; Bruner et al., 2011; Rossing et al., 2015). Contextual factors such as cultural differences intra- and inter-regional communities within European countries, distances among communities, the number and quality of sport facilities, the number of experts or the competitive depth of a given sport, represented important obstacles that could further justify this inconsistency in European results. For this reason, to proceed with the *second hypothesis* of this study, we chose to select only the players born in the USA (*n* = 1,389).

### Statistical Analysis

In accordance to the statistical procedures used in previous studies examining the RAE and/or the birthplace (Hancock et al., 2018), we analyzed the differences among the distributions of observed and expected percentages.

After analyzing the variables for skewness, kurtosis, normality distribution through Kolmogorov–Smirnov test, and the equality of variances by Levene test, parametric or nonparametric statistical tests were used when appropriated assuming a confidence level of 0.05. A chi-square test χ^2^ was used to analyse de distribution of dates of birth across the 4 quartiles Q1, Q2, Q3, and Q4. Odds ratios (OR) and 95% confidence intervals (CI) were calculated to determine the odds of a player born in Q1 (January–March) playing in the NBA compared to the odds of a player born in Q2, Q3, and Q4. An OR with CI limits (lower and upper) higher than 1 indicates that a disproportionately high number of NBA-drafted players were observed in each quartile compared to that expected due to the general population distribution. Correspondingly, an OR with CI lower and upper limits lower than 1 indicates that a disproportionately low number of drafted players was observed. RAE were compared among the different groups (quartiles) using Wilcoxon and Mann–Whitney *U* tests.

Similar statistical procedures were used to analyse the differences among the distributions of observed and expected communities' size categories. Thus, χ^2^, ES, OR, and 95% CI were calculated to determine the odds of a player born in a smaller community, i.e., <50,000 inhabitants playing in the NBA compared to the odds of a player from the remaining categories. For analysing RAE and birthplace effects, we selectively used data from USA's 1990 Data and Statistics and Europe's 2000 Eurostat, as they were the closest possible to the average year of birth of the players included in our selective sample.

Cluster analysis was performed using Ward's method—squared Euclidian distance as a distance measure—using RAE and birthplace effects quartiles and communities' size. The χ^2^ test was used to examine between-conditions differences in terms of RAE and birthplace effects. Standardized residuals (e) were used to determine which variable(s) in each category contributed most to the value of χ^2^. Cells which contained values of standardized residual that were higher than 1.96 (e> 1.96), were considered influent for the model.

Univariate analysis of variance (ANOVA) test and Tukey's *post-hoc* test was used in conjunction to examine the differences between clusters. Statistical analyses were performed using SPSS v.25 software (Inc., Chicago, IL, USA) and significant level was set at *p* < 0.05.

## Results

Table 2 presents the frequency and percentage distribution of the drafted players' birth quartiles. The χ^2^ analysis confirmed a biased distribution both for USA and European players (*p* = 0.005 and *p* = 0.028, respectively). For American players, the distribution of the players' birthdates in Q1 (28.2%) was higher than those in Q2 (23.9%), Q3 (24.7%), and Q4 (23.3%). The analysis of the RAE for players born in European countries revealed similar results, however the higher percentage was observed in Q2 (32.1%). For USA players, the likelihood of player born is Q1 being selected to play in the NBA yielded an OR higher than 1 [OR = 1.236, CI (1.044–1.465)]; similar results were found for European players, as Q1 and Q2 presented an OR higher than 1 [OR = 1.148, CI (0.760–1.734); OR = 1.378, CI (0.924–2.051), respectively].

**Table 2:** Descriptive and inferential analysis for dates of birth for USA and European players.

**Birth quarter**	**USA**						**Europe**					
	**CDC^**a**^**	**%**	**OR**	**CI**	**χ^2^**	***p***	**EU^**b**^**	**%**	**OR**	**CI**	**χ^2^**	***p***
Q1 (Jan–Mar)	24.10	28.2 (392)	1.236	1.044–1.465	12.888	0.005*^#^	24.36	27.0 (64)	1.148	0.760–1.734	9.13	0.028^*$*^
Q2 (Apr–Jun)	24.96	23.9 (332)	0.944	0.794–1.122			25.54	32.1 (76)	1.378	0.924–2.051		
Q3 (Jul–Sept)	26.43	24.7 (343)	0.913	0.770–1.083			25.85	19.4 (46)	0.690	0.447–1.065		
Q4 (Oct–Dec)	24.60	23.3 (324)	0.931	0.782–1.108			24.25	21.5 (51)	0.855	0.556–1.314		

a *Data collected from USA's 1990 Data & Statistics*.

b *Data collected from Europe's 2000 Eurostat*.

* *Significant difference (p < 0.05) between Q1 and Q2*;

# *Significant difference (p < 0.05) between Q1 and Q4*;

$ *Significant difference (p < 0.05) between Q2 and Q3*.

*Legend: OR, odds ratio; CI, confidence interval*.

The distribution of the USA and European players across different communities' size categories are displayed in Table 3. Results confirmed a different distribution in the categories between the USA and European census and the players selected for the NBA Draft. Smaller categories are over-represented in the drafted players, especially with less than 100,000 inhabitants, both for USA and European draftees. Moreover, communities with a population above 1,000,000 yielded an OR lower than 1. This means that the likelihood for players born in big city to be selected and play in the NBA was lower than for players born in a smaller community. In addition, the likelihood for players who were born in a smaller city (i.e., 50,000–99,999) was higher than the remaining categories [USA OR = 2.109, CI (1.632–2.726); EU OR = 15.103, CI (10.736–21.248)].

**Table 3:** Descriptive and inferential analysis for places of birth for USA and European players.

**Population size**	**USA**				**Europe**			
	**CDC^**a**^**	**%**	**OR**	**CI**	**EU^**b**^**	**%**	**OR**	**CI**
>5,000,000	23.19	0.7	0.024	0.013–0.045	0.92	0.7	0.759	0.165–3.496
2,500,000–4,999,999	13.91	9.6	0.657	0.520–0.830	2.30	4.2	1.862	0.987–3.513
1,000,000–2,499,999	15.65	8.5	0.501	0.395–0.635	11.07	9.2	0.796	0.511–1.242
500,000-−999,999	10.49	15.6	1.577	1.260–1.974	14.61	10.9	0.715	0.475–1.076
250,000–499,999	9.12	11.9	1.346	1.054–1.718	14.54	12.6	0.847	0.577–1.244
100,000–249,999	8.94	15.7	1.897	1.501–2.397	10.23	16.7	1.759	1.251–2.475
50,000–99,999	0.7	13.7	2.109	1.632–2.726	1.31	16.7	15.103	10.736–21.248
<50,000	18.00	24.4	1.471	1.224–1.767	45.02	26.8	0.447	0.335–0.596

a *Data collected from USA's 1990 Data & Statistics*.

b *Data collected from Europe's 2000 Eurostat*.

*Legend: OR, odds ratio; CI, confidence interval*.

**Figure 3:**
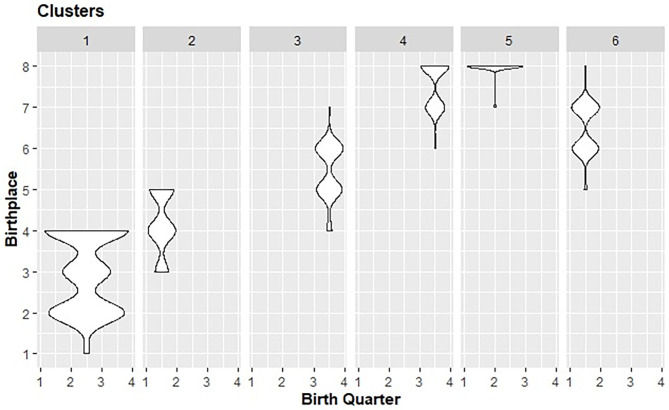
Violin plots including RAE and BPE density distribution amongst distinct clusters. Note: Birthplace categories should be interpreted as (1) more than 5,000,000 million of inhabitants; (2) between 2,500,000 and 4,999,999; (3) 1,000,000–2,499,000; (4) 500,000–999,999; (5) 250,000–499,999; (6) 100,000–249,999; (7) 50,000–99,999; (8) less than 50,000 inhabitants. Birth quarters should be interpreted as (1) Janury–March; (2) April–June; (3) July–September; (4) October–December.

The cluster analysis established different levels of interaction in terms of birth quartiles, communities' size categories and career stats profiles. Table 4 and Figure 3 displays these interactions where it stands out (i) clusters corresponding to the players born in the bigger communities relate mainly with relatively younger players; (ii) clusters that correspond to players born in smaller communities integrated the relatively older players; (iii) no differences were found in the career stats.

**Table 4:** Descriptive and inferential analysis for career performance, RAE and birthplace for USA players.

		**Cluster 1**	**Cluster 2**	**Cluster 3**	**Cluster 4**	**Cluster 5**	**Cluster 6**	***p***
		***n* = 375**	***n* = 184**	***n* = 189**	***n* = 172**	***n* = 247**	***n* = 222**	
Career performance	%GS	32.6 ± 30.9	32.9 ± 29.5	32.8 ± 31.1	34.3 ± 30.4	30.8 ± 29.6	33.3 ± 31.1	0.924
	MPG	18.3 ± 9.0	18.5 ± 8.4	18.1 ± 9.0	18.5 ± 8.6	18.3 ± 8.2	19.2 ± 8.7	0.880
	PPG	18.0 ± 4.8	17.3 ± 4.8	18.3 ± 5.8	17.7 ± 6.5	17.5 ± 4.7	18.7 ± 7.5	0.159
	Rebounds	8.1 ± 3.5	8.5 ± 3.7	8.7 ± 3.7	8.4 ± 3.9	8.3 ± 3.4	8.0 ± 3.6	0.429
	Assist:TO	1.4 ± 0.8	1.3 ± 0.7	1.2 ± 0.7	1.3 ± 0.7	1.4 ± 0.7	1.4 ± 0.8	0.127
	PPIndex	41.7 ± 8.8	40.7 ± 9.4	42.3 ± 11.1	41.3 ± 12.0	40.8 ± 9.0	42.4 ± 11.8	0.478
Birth quarter	Q1 (Jan–Mar)	−6.3	**12.2**	−7.3	−7.0	**3.2**	**6.5**	
	Q2 (Apr–Jun)	1.3	0.0	−6.7	−6.4	**2.4**	**7.6**	
	Q3 (Jul–Sep)	**3.5**	−6.7	**8.3**	−0.4	1.7	−7.4	
	Q4 (Oct–Dec)	**2.0**	−6.5	**6.3**	**14.5**	−7.6	−7.2	
Birthplace	>5,000,000	**4.4**	−1.2	−1.2	−1.1	−1.3	−1.3	
	2,500,000–4,999,999	**16.3**	−4.2	−4.3	−4.1	−4.9	−4.6	
	1,000,000–2,499,999	**8.8**	**5.4**	−4.0	−3.8	−4.6	−4.4	
	500,000–999,999	**11.8**	**7.5**	−5.4	−5.2	−6.2	−5.9	
	250,000–499,999	−6.7	**11.9**	**13.9**	−4.5	−5.4	−5.2	
	100,000–249,999	−7.5	−5.3	**13.3**	−5.1	−6.1	**13.2**	
	50,000–99,999	−7.2	−5.0	−5.1	**11.0**	−5.8	**14.9**	
	<50,000	−9.6	−6.7	−6.8	**8.1**	**23.9**	−7.4	

*Legend: %GS, % of game starts; MPG, minutes per game; PPG, points scored per game; Steal:TO, ratio steal per turnover; Assist:TO, ratio assist per turnover; PPIndex, player performance index. Data is presented as M ± SD. Bold corresponds to cells which contained values of standardized residual that were higher than 1.96 (e > 1.96)*.

## Discussion

The main aims of this study were two-fold: (1) to inspect separately for the RAE and birthplace effects for players born in the USA and in the European countries; (2) to explore the interaction among these contextual factors and the long-term effect of both these factors in the players' career performance. We found a biased distribution of the birth quartiles, as the number of players born in Q1 or Q2 was higher than that of those born in Q3 or Q4 both for those born in the USA and born in European countries. These results are consistent with previous research confirming an advantage in favor of the earlier born players emerging from the athletic programs of colleges and universities in the United States of America, Canada and European clubs.

The NBA is an extremely competitive professional league, which makes highly demanding the process of preparing and selecting players. The franchises have to choose the best players based on their abilities and potential, but must be able to predict the level of future performance. Thus, the selection process (i.e., the NBA Draft) is highly conditioned by the players' performance and favors the selection of those athletes who were born in the first months of the year and that will have a greater impact on the team's performance (De la Rubia et al., 2020a). In the case of basketball, the presence of the RAE still manifests itself around 20–22 years of age, at the same age category where NBA Draft take place (De la Rubia et al., 2020a). Therefore, there may be a ripple effect that benefits the selection of relatively older athletes, despite it still unknown how it affects the long-term performance of the athlete, that is, in his sports career.

In sports like basketball, the “maturation-selection hypothesis” applies, i.e., relatively elder players are naturally heavier, taller, stronger, and faster than other (Baker and Logan, 2007; Zaric et al., 2020), and can simultaneously benefit from an advantage resulting from being born in an earlier month, but also from an early maturation, to have better opportunities (Arede et al., 2019b). This seems to make sense, given that the anthropometric aspects important factor for the performance in basketball (Teramoto et al., 2017; Zaric et al., 2020). In fact, the results obtained during the annual event hosted by NBA dedicated to assess the players with greatest potential (i.e., NBA Draft Combine) have shown that length-size aspects, such as height, body mass, wingspan, and hand dimensions have the greatest positive correlation with on-court performance in the NBA, in the short and medium term (Teramoto et al., 2017). However, based on present results, we may hypothesize that this initial physical/anthropometric advantage (i.e., the NBA Draft) is circumstantial and does not remain across the professional career; instead, relatively younger players born in Q3 e Q4 (USA) and Q3 (European countries), i.e., may overcome that disadvantage and gradually develop the physical, technical, tactical, and mental skills required to succeed in the NBA (Sampaio et al., 2015). These results are particularly important given that, for the first time, they confirm that in a long-term development perspective, elite basketball seems not to favor the relatively older players.

Previous knowledge about players' potential as well as coaches and scouts' biases, should be considered as another possible reason for our results (Till and Baker, 2020). For example, Mann and van Ginneken (2017) reported that the selection bias could be reduced when scouts watched junior soccer games analyzing the shirt numbers corresponded to the RAE of the players. The authors concluded that the selection bias associated with the RAE can be reduced, if information about age is presented appropriately. Although the RAE investigation is extensive (see De la Rubia et al., 2020b for extensive review on the RAE), many coaches and scouts are not yet fully informed about this phenomenon, and therefore, further investigation and an effective bridge between science and practice is required (Olswang and Prelock, 2015).

The results of our study are consistent with previous literature on birthplace effects, showing that the place where the athlete is born does not hinder the possibility of attaining elite performance (MacDonald et al., 2009b). Interestingly, a great number of players drafted to the NBA born in smaller cities (<50,000; 24.4 and 26.8% for USA and European players, respectively). The same trend has been observed in different professional sports, such as ice hockey, baseball, golf, football, and soccer (Côté et al., 2006; MacDonald et al., 2009a,b; Wattie et al., 2018).

Globally, evidence from our study confirm that smaller communities can match medium and large communities in early opportunities for talent development. While the notion of the “small town effect” has been examined recently, most of the researchers did not find empirical evidence to support that concept in pathways to sport expertise (Pennell et al., 2017). Several explanations for the contribution of communities of this size to the development of talent in sport have already been proposed, among which we underline larger social support by their peers, families, enabling them to develop their skills and also an increased perception of safety in open-spaced areas (MacDonald et al., 2009a). Moreover, these smaller population areas can also foster more supportive relationships between coaches and athletes, and even with the social context that surrounds the sport that favor the feeling of belonging as well as a much more positive learning context. Under such circumstances, young athletes are more likely to develop a positive self-concept and acquire the necessary motivation for a long-term and positive involvement in sport (Moesch et al., 2013). Additional benefits have been associated to smaller communities. In fact, children seem to allocate more time to the multi-faceted process of learning the sport skills that are relevant to their specific sport or being able to practice sport in a less structured environment (“deliberate play”), in such a way that it favors the development of tactical creativity and perceptual and decision-making capacity (Berry et al., 2008; Greco et al., 2010; Memmert et al., 2010; Ford et al., 2011; Leite and Sampaio, 2012; Arede et al., 2019a). Simultaneously, playing more often against the same opponents can have a beneficial effect. In a team sport like basketball, having prior knowledge about your opponent strengths and weaknesses, can help the player to be more focused on improving and recognizing offensive and defensive game patterns to explore.

The second aim of the study was to examine the potential interaction between RAE and birthplace in players born in players born in the USA players. The only available study that hypothesized about this interaction was done by Bruner et al. (2011), however, no evidence of interaction was found. Interestingly, our data supported the theoretically driven hypothesis that USA players born in the bigger communities were relatively younger; players born in smaller communities integrated relatively older players; and NBA career performance was similar irrespectively of this interaction. According to Barker's “theory of behavior settings” (Barker, 1968) the number of people in a behavior setting will influence an individual's behavior. Thus, it could be expected that situations with fewer (i.e., smaller) or more than (i.e., bigger) the optimal number of participants needed to complete a task will result in different experiences for individuals involved. Thus, in light of our results, it may be hypothesized that smaller communities may be more likely to promote greater youth developmental opportunities for relatively older athletes, while bigger communities may be more appropriate environment for relatively younger athletes. Smaller cities seem to provide an environment in which, despite infrastructural resources, the number of teams and players being smaller, the need to select athletes based on their skill and maturation levels is higher. Consequently, RAE may be reduced or even removed in higher cities where these variables are not an issue, and therefore, seem to be more appropriate to further develop talent. Despite the interest and usefulness of these results for talent development, future studies are warranted to test this interaction.

The interaction little explains about the NBA career, however their experiences until getting there may have been influenced by these two factors. Both players who came from bigger cities or born in last quarters can be exposed to adverse experiences in their development, due to their disadvantageous status (McCarthy and Collins, 2014; McCarthy et al., 2016; Collins and Macnamara, 2017). However, they can perform a greater effort in the learning process, but also develop psychological skills becoming more able to overcome difficulties (Cobley et al., 2009; Gibbs et al., 2012), what can become a long-term advantage. The possible additional pressure experienced by the player born at the end of the year, can be an advantage in the long term. Undoubtedly, the challenge would be to know which of these two types of populations is likely to have a higher performance in the long term or to have longer and more successful sports careers.

The results of this study provide compelling evidence that contextual factors influence the likelihood to being selected to play in NBA. However, we must address potential limitations of our study, which must be acknowledged. First, the NBA Draft is an highly selective event, in which teams try to address their particular needs and not only look into de best prospect available in the board, regardless of the RAE and birthplace effects (Zhang et al., 2018). This logic is also observed in elite football that favors late maturing boys succeed in achieving elite level, despite not having benefited from a maturation bias through development process (Ostojic et al., 2014). That said, as the level of performance increases, performance in the short and medium term, considering the needs of the team can be decisive, more than other contextual factors.

Second, a challenge in birthplace effects studies is the analysis of population categories. We followed previous categorization used in studies with North-American athletes, but in each state, there are significant variations not only in terms of communities' sizes and also in population density. Researchers should be encouraged to explore methods by which to limit such variability, enabling stronger within-category consistency. This sizeable variation is even more critical in European countries, however, while we admit this limitation, we believe that this could trigger further studies that could highlight differences within European sport policies and youth development programs.

Additionally, it is important to acknowledge that birthplace does not always coincide with the athlete's place of development and that athletes may migrate among locations. For example, athletes born in small rural communities may move to larger urban centers during their childhood (Schorer et al., 2010). Another limitation may be the fact that this research is based on the interaction of the RAE and the birthplace effects in the short term, at a very specific moment such as the choice of the NBA Draft, but it would probably be more interesting to know the interaction of these effects in the long term, throughout the athlete's career, to know which players can have a longer sports career or have a higher performance.

## Conclusions

The results of the current study confirm a biased distribution for players born in the initial months in each selection year against those born in smaller cities in the NBA. Evidence from this study is consistent with previous literature providing important considerations about the influence of contextual factors in achieving expertise in sport. Understanding the interaction between RAE and birthplace effects, can help researchers and practitioners on how to design sports systems and practice approaches to help nurture talent. Creating the most favorable environment for talent development is highly complex, due to performance multidimensionality. Delaying the selection of players until ages like those used in NBA Draft (mostly after 19–20 years) is a suggestion systematically repeated by experts, but at the same time we must continue on a path to discover and integrate new variables in performance analysis. The results of this study represent an important contribution in this field since this interaction between contextual factors hasn't been described in the literature and in addition to helping us to know better the system, it can contribute to increase retention and improve coaches decision making.

## Data Availability Statement

The raw data supporting the conclusions of this article will be made available by the authors, without undue reservation.

## Ethics Statement

The studies involving human participants were reviewed and approved by University of Trás-os-Montes and Alto Douro. Written informed consent for participation was not required for this study in accordance with the national legislation and the institutional requirements.

## Author Contributions

NL, JA, and XS: conceptualization. NL, JA, and XS: investigation. NL and JA: methodology. NL, XS, and JA: data curation. NL, JA, XS, JC-G, and AL: formal analysis. NL and JA: software. NL and XS: writing—original draft. NL, JA, JC-G, and AL: writing—review and editing: All authors contributed to the article and approved the submitted version.

## Conflict of Interest

The authors declare that the research was conducted in the absence of any commercial or financial relationships that could be construed as a potential conflict of interest.
